# Global trends and research hotspots in coronary revascularization for ischemic heart disease: a bibliometric analysis (2005–2024)

**DOI:** 10.1186/s13019-026-03937-w

**Published:** 2026-03-03

**Authors:** Jia-ming He, Ying-qin Kong, Ding-Jun Wang, Xin-hai Feng, Yuhao Dong, Zhe-hao Yin, Dong Zhou

**Affiliations:** 1 Department of Cardiovascular Surgery, Xinqiao Hospital, Third Military Medical University (Army Medical University), Chongqing, 400037 China; 2 Department of Pathology, Xinqiao Hospital, Third Military Medical University (Army Medical University), Chongqing, 400037 China; 3 Department of Thoracic Surgery, Xinqiao Hospital, Third Military Medical University (Army Medical University), Chongqing, 400037 China

**Keywords:** Ischemic cardiomyopathy, Coronary artery disease, Revascularization, Percutaneous coronary intervention, Coronary artery bypass grafting

## Abstract

**Objective:**

To provide the first comprehensive bibliometric analysis of global scientific literature on coronary revascularization over the past two decades, mapping publication trends, influential contributors, collaborative networks, and the evolution of research hotspots.

**Methods:**

A systematic search of the Web of Science Core Collection (WoSCC) was conducted for English-language articles and reviews on CAD revascularization published between 1 January 2005 and 30 September 2024. Bibliometric analyses were performed using Microsoft Excel, the *bibliometrix* R package, CiteSpace, and VOSviewer. Key parameters included association strength (VOSviewer), Pathfinder pruning and Latent Dirichlet Allocation (CiteSpace), with core indicators (MCP ratio, H-index, link strength) integrated to ensure rigor. A PRISMA-style flowchart detailed the literature selection process.

**Results:**

The analysis included 372 publications from 55 countries. European countries led in publication volume, while the United States demonstrated high citation impact. Leading institutions included Erasmus MC, Imperial College London, and the Cardiovascular Research Foundation. Serruys PW and Stone GW emerged as influential authors, with *EuroIntervention*, *The Lancet*, and *Circulation* as core journals. Keyword and citation analyses identified three dominant themes: comparative effectiveness of PCI vs. CABG, minimally invasive revascularization techniques, and technological innovations (e.g., OCT/IVUS, bioadaptable stents). Temporal evolution revealed a shift toward personalized strategies and AI-assisted intervention*.*

**Conclusion:**

This study provides the first comprehensive bibliometric synthesis of CAD revascularization research, highlighting global productivity, collaboration, and thematic trends from device optimization to personalized, strategy-oriented care. The findings offer a structured reference for clinicians, researchers, and policymakers to navigate the field’s intellectual foundations and emerging directions.

**Supplementary Information:**

The online version contains supplementary material available at 10.1186/s13019-026-03937-w.

## Introduction

Coronary artery disease (CAD) remains the leading cause of morbidity and mortality worldwide and is the primary etiology of heart failure in developed nations [1]. Revascularization—comprising percutaneous coronary intervention (PCI) and coronary artery bypass grafting (CABG)—constitutes the cornerstone of therapeutic management for obstructive CAD, aiming to restore myocardial perfusion, alleviate symptoms, and improve prognosis [2, 3]. Over the past two decades, the field has undergone transformative evolution, marked by landmark clinical trials (e.g., SYNTAX, FAME, EXCEL, ISCHEMIA, REVIVED-BCIS2) and significant technological advancements, particularly in drug-eluting stent (DES) design, intravascular imaging, and physiological guidance [4, 5]. This prolific scientific output has generated a vast and complex body of literature, reflecting shifting paradigms in patient selection, procedural strategy, and long-term management.

In recent years, bibliometric analysis has emerged as a powerful tool to map the structure, dynamics, and evolution of scientific fields. By analyzing publication patterns, citation networks, author collaborations, and keyword co-occurrences, bibliometrics provides a macroscopic overview of research productivity, intellectual influences, and emerging trends [6]. Several bibliometric studies have been successfully applied to adjacent cardiovascular domains, offering valuable insights. For instance, analyses have mapped global trends in anesthesia for CABG surgery [7], PCSK9 research [8], and coronary microvascular dysfunction [9]. These studies employ established methodologies—using databases such as Web of Science, and visualization tools like VOSviewer and CiteSpace—to decode the knowledge architecture of their respective fields.

Despite the critical clinical importance and high research activity surrounding coronary revascularization, a dedicated, comprehensive bibliometric synthesis of this specific domain is notably absent. Existing reviews and meta-analyses predominantly focus on clinical outcomes, device comparisons, or guideline updates [10, 11], but none have systematically employed bibliometric methods to visualize the global research landscape, identify key contributors, trace thematic evolution, and forecast future directions over an extended period.

Therefore, this study aims to fill this gap by conducting the first integrated bibliometric analysis of global scientific literature on coronary revascularization for ischemic heart disease from 2005 to 2024. Specifically, our objectives are to: (1) quantify publication trends and geographic distributions; (2) identify leading institutions, authors, and core journals; (3) map collaboration and co-citation networks; (4) detect research hotspots and trace their temporal evolution using keyword burst detection and timeline visualization; and (5) interpret these findings within the context of clinical advancements and trial evidence. By doing so, this analysis will provide a unique evidence map for clinicians, researchers, and policymakers, highlighting established knowledge cores, current frontiers, and potential avenues for future investigation in this dynamically evolving field.

## Materials and methods

### Data sources and searches

A systematic literature search on coronary revascularization in ischemic heart disease was performed on 1 October 2024 using the Web of Science Core Collection, with the Science Citation Index Expanded and the Social Sciences Citation Index as citation sources. An advanced search strategy was applied with the following terms: TS = (“myocardial ischemia” OR “coronary artery disease” OR “ischemia”) AND TS = (“revascularization”). The initial search yielded 1,235 records. To ensure focus and relevance, the dataset was refined according to the following criteria: (1) publication period between 1 January 2005 and 30 September 2024; (2) document type limited to articles and reviews; (3) language restricted to English; (4) exclusion of duplicates (identified and removed using WoSCC’s built-in duplicate detection tool and manual cross-verification); (5) exclusion of early access publications not yet fully indexed; (6) exclusion of non-research articles (e.g., editorials, letters to the editor, case reports); and (7) exclusion of studies outside the scope of CAD and coronary revascularization (e.g., revascularization for non-coronary vascular diseases). A PRISMA-style flow diagram (Supplementary Figure S1) details the entire selection process, including the number of records excluded at each stage. As all data were obtained from publicly available databases, ethical approval was not required.

### Data analysis

Data compilation and generation of annual publication histograms were conducted using Microsoft Excel 2021. Comprehensive bibliometric analysis and scientific mapping were primarily conducted using the bibliometrix R package (v4.4.1) [12]. This open-source tool was employed to calculate standard bibliometric indicators, including: total publications (TP) and citations (TC) for countries, institutions, authors, and journals; average citations per publication; the Multiple Country Publications (MCP) ratio to assess international collaboration intensity; and author-level metrics (H-index) where applicable. The bibliometrix package was also used to generate the global geographical distribution map of publications.

To delve deeper into the intellectual structure and dynamic evolution of the field, two specialized software tools were utilized:

1. VOSviewer (version 1.6.20): This software was used to construct and visualize bibliographic networks based on co-authorship (countries, institutions, authors), co-citation (authors, references, journals), and keyword co-occurrence [6]. For network construction in VOSviewer, the association strength method was selected for normalization, which is the default and recommended setting for creating robust and interpretable maps in bibliometric studies [6, 7]. Minimum threshold criteria were set for each network type (e.g., a minimum number of documents for a country to be included) to ensure clarity. In the resulting maps, node size represents the weight of the item (e.g., publication count, citation count), link thickness indicates the strength of the relationship (e.g., collaboration frequency, co-citation strength), and colors denote clusters of closely related items.
2. CiteSpace (version 6.4.R6, 64-bit): This tool was applied for burst detection analysis (identifying sharp increases in citations or keyword frequency over time), timeline visualization of keyword clusters to trace thematic evolution, and reference clustering to identify foundational knowledge bases [13]. In CiteSpace, the log-likelihood ratio (LLR) algorithm was used for cluster labeling to ensure thematic clarity, and pathfinder network pruning was applied to simplify the network while retaining its salient structure, following established methodologies [13, 14].

In constructing productivity rankings (e.g., for countries and institutions), we adhered to the standard bibliometric practice of attributing publications and citations to all listed authors and their affiliations. We acknowledge that the presence of highly prolific authors can influence such rankings. To ensure transparency and to allow readers to contextualize the findings, we have explicitly identified these key contributors in the results (e.g., Professor Serruys PW and Stone GW) and report their individual productivity metrics separately. The primary goal of our country/institution analysis is to map the observable scientific output and collaboration structures as they exist in the published literature, which inherently includes the outsized impact of leading figures. Our subsequent thematic analyses (keyword co-occurrence, citation bursts, timeline views) are based on the entire corpus and are designed to reflect the intellectual content and evolution of the field, which is less susceptible to distortion by individual author volume.

## Results

### Publication trends and growth trajectory

**Fig. 1:**
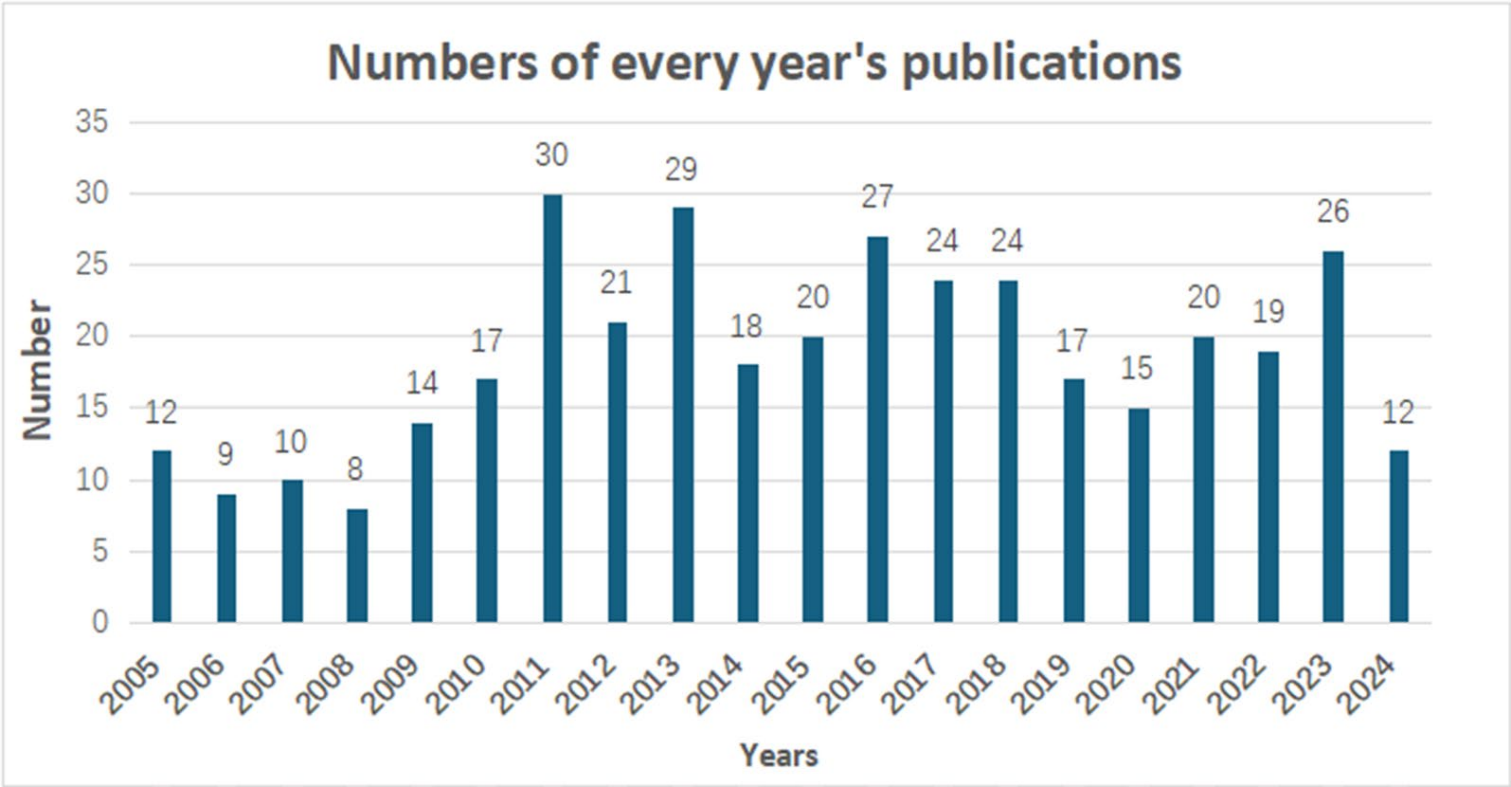
Annual trends of global publication outputs in the research

A total of 372 documents on ICM revascularization were retrieved from the WoSCC database, comprising 307 original articles and 65 reviews. Fig 1 depicts the annual publication volume from 2005 to 2024. Following a period of growth, output peaked at 30 publications in 2011, coinciding with the dissemination of major trial results (e.g., SYNTAXES, FREEDOM) and technological debates surrounding first- and second-generation drug-eluting stents (DES). Subsequently, annual publication counts stabilized at approximately 20, indicating a mature yet consistently active field, sustained by successive iterations of device technology and ongoing comparative effectiveness research (e.g., ISCHEMIA, EXCEL, NOBLE trials).

### Geographic distribution and collaborative networks

**Fig. 2:**
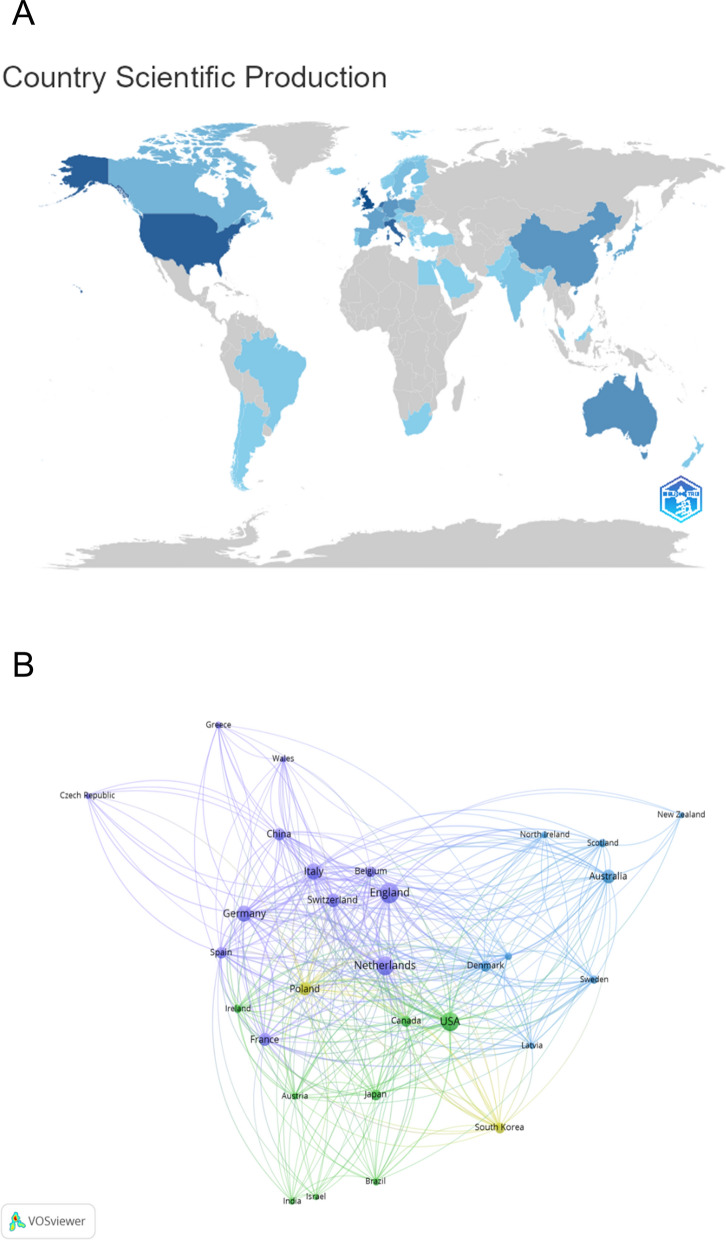
Geographical distribution characteristics of global publications and analysis of international collaboration networks of the research. (**A**) Geographical distribution of countries in terms of publications. (**B**) Network mapping of international collaboration

Fifty-five countries and regions contributed to the literature. As presented in Table 1 and Supplementary Figure S2, European nations dominated in terms of volume, with the United Kingdom leading (104 publications, 28.0%), followed by the Netherlands (84, 22.6%) and Italy (60, 16.1%). The United States ranked third in output (83) but demonstrated the highest average citation rate (66.34), underscoring the substantial global impact of its research. Analysis of the Multiple Country Publications (MCP) ratio revealed that international collaboration was most frequent among European countries and between the US and European partners. Fig 2A and 2B isualizes the international co-authorship network, with the UK, USA, Netherlands, Germany, and Switzerland forming the most interconnected and central nodes, highlighting a transatlantic axis of research collaboration.

**Table 1:** The top 10 productive countries in the research field

Rank	Countries	Publications	Citations	Citations per publication
1	England	104	5847	56.22
2	Netherlands	84	3726	44.35
3	USA	83	5507	66.34
4	Italy	60	2725	45.41
5	Germany	56	2887	51.55
6	Switzerland	40	2129	53.23
7	Australia	39	2358	60.46
8	Poland	35	971	27.74
9	France	32	1712	53.50
10	Spain	27	1558	57.7

### Institutional productivity and influence

**Fig. 3:**
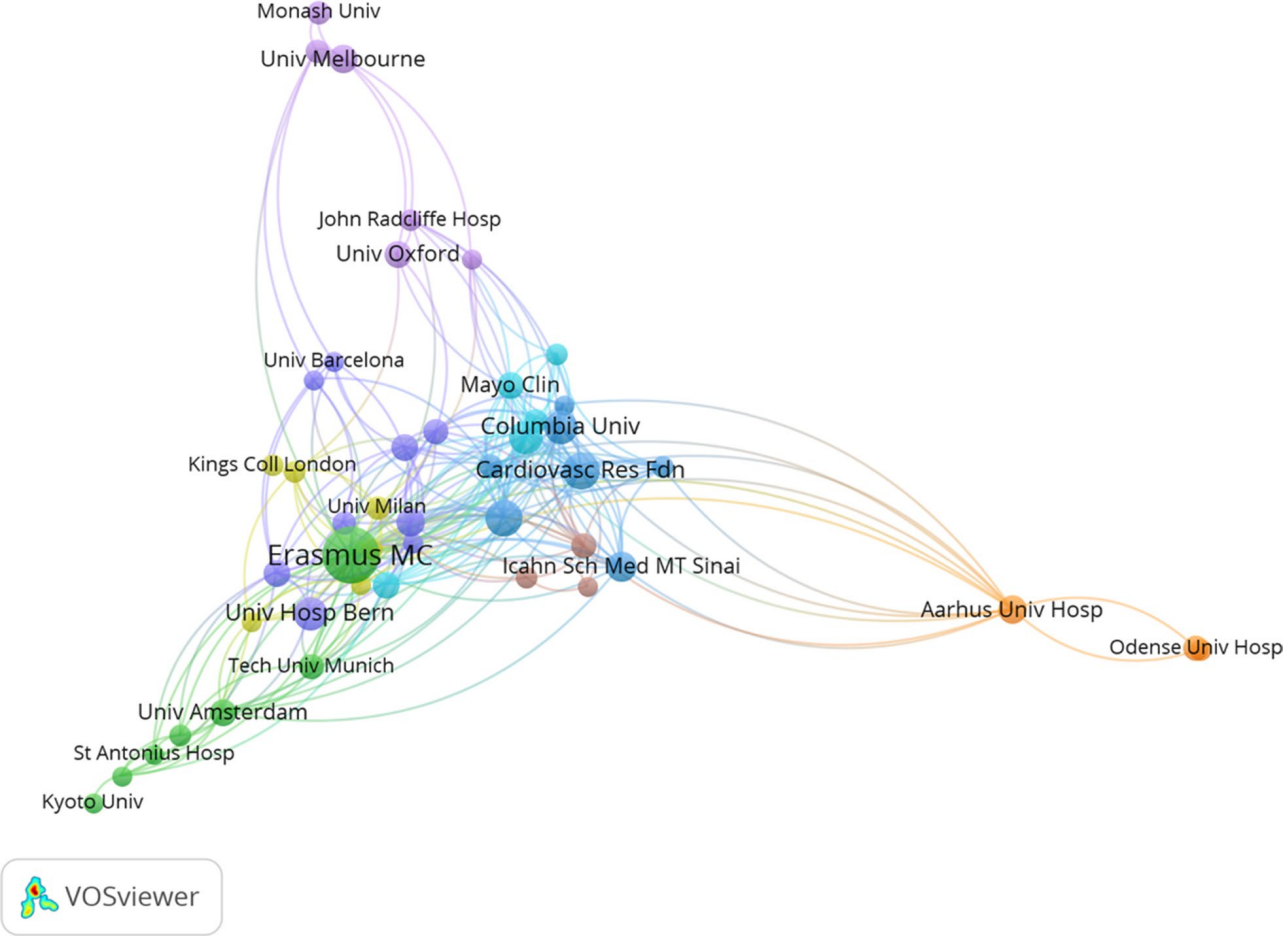
The co-authorship map of organizations with a minimum of 5 publications

VOSviewer analysis identified 1,015 institutions engaged in coronary revascularization research. Table 2 lists the top 10 most productive institutions. Erasmus MC (Netherlands) was the most prolific (36 publications), while Imperial College London (UK) and the Cardiovascular Research Foundation (USA) exhibited higher average citation rates per paper. The institutional co-authorship network (Fig. 3) reveals distinct clusters centered around these leading hubs. Erasmus MC and the Cardiovascular Research Foundation serve as pivotal bridges connecting European and North American research consortia, respectively, reflecting their central roles in large, multinational clinical trials.

**Table 2:** The top 10 productive institutions for the research

Rank	Institutions	Publications	Citations	Country	Citations per publication
1	Erasmus MC	36	719	Netherlands	19.97
2	Imperial College London	16	1111	England	69.44
3	Cardiovasc Res Fdn	16	1133	USA	70.81
4	Univ Hosp Bern	14	712	Switzerland	50.86
5	Erasmus University	13	1695	Netherlands	130.38
6	Columbia University	13	948	USA	72.92
7	The Icahn School of Medicine at Mount Sinai	11	1025	USA	93.18
8	Aarhus Univ Hosp	10	1245	Denmark	124.50
9	The University of Catania	10	516	Italy	51.60
10	The University of Melbourne	10	195	Australia	19.50

### Authors and intellectual leaders

**Fig. 4:**
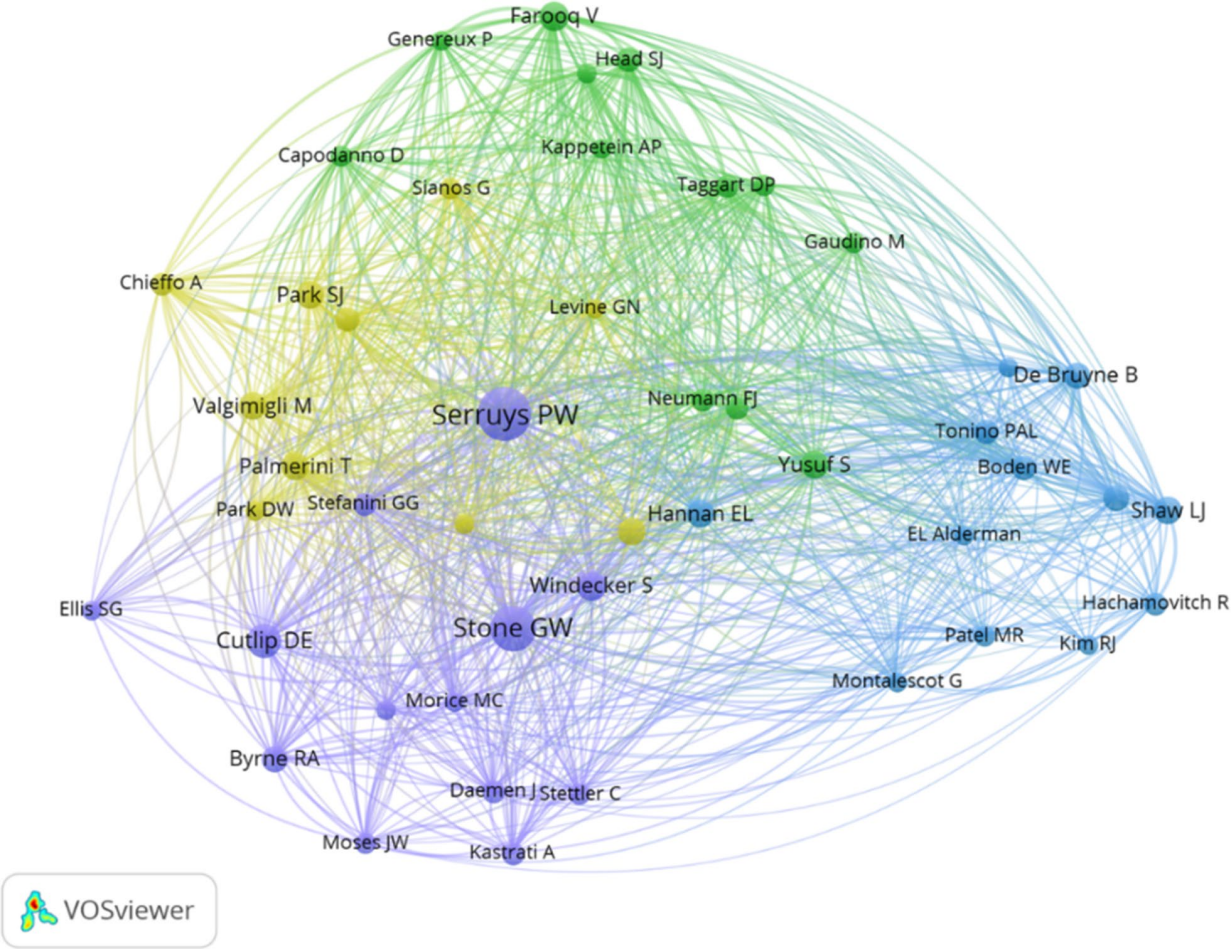
Cocited authors analysis map with a minimum of 20 cocitations. ​

A total of 2,604 authors contributed to ICM revascularization research over the past two decades. Table 3 and Supplementary Figure S3A present the 10 most prolific authors, with notable contributions from the UK and Ireland. Professor Serruys PW emerged as the most influential author, with 33 publications and 2,153 citations. Supplementary Figure S3B maps author collaboration networks, showing fragmented clusters that highlight opportunities for stronger interdisciplinary and inter-institutional cooperation. Co-citation analysis (Fig. 4) further confirmed Serruys PW and Stone GW as the most frequently cited authors, establishing them as key opinion leaders in the field.

**Table 3:** The most productive authors in the research field

Rank	Authors	Documents	Citations	Average Citation/Publication	Country
1	Serruys PW	33	2153	65.24	Ireland
2	Morice MC	14	1448	103.43	France
3	Windecker S	14	967	69.07	Switzerland
4	Yoshinobu Onuma	11	822	74.73	Ireland
5	Kappetein AP	10	1326	132.60	Netherlands
6	Stone GW	10	802	80.20	USA
7	Antonio Colombo	10	908	90.80	Italy
8	Mohr FW	7	1219	174.14	Germany
9	Farooq V	7	860	122.86	England
10	William Wijns	7	664	94.86	Ireland

The analysis revealed that a subset of authors, most notably Professor Patrick W. Serruys and Dr. Gregg W. Stone, contributed a substantial number of publications and citations (Table 3). This concentration of output is a known phenomenon in fast-moving, trial-intensive clinical fields like interventional cardiology, where certain research groups and thought leaders play a defining role in major clinical trials and consensus documents. Their high productivity naturally influences the aggregate publication counts of their affiliated countries (e.g., the Netherlands, USA) and institutions (e.g., Erasmus MC, Cardiovascular Research Foundation). Our ranking tables (Tables 1, 2, 3) should therefore be interpreted as reflecting the total, observed scientific landscape, which is shaped by both broad-based research activity and the concentrated efforts of pivotal individuals. The thematic and evolutionary analyses presented in the following sections (e.g., Figs. 6 and 7**)**, which are based on keyword and citation patterns across the entire dataset, provide a complementary perspective that highlights dominant research concepts beyond individual authorship.

### Core journals and knowledge dissemination

**Fig. 5:**
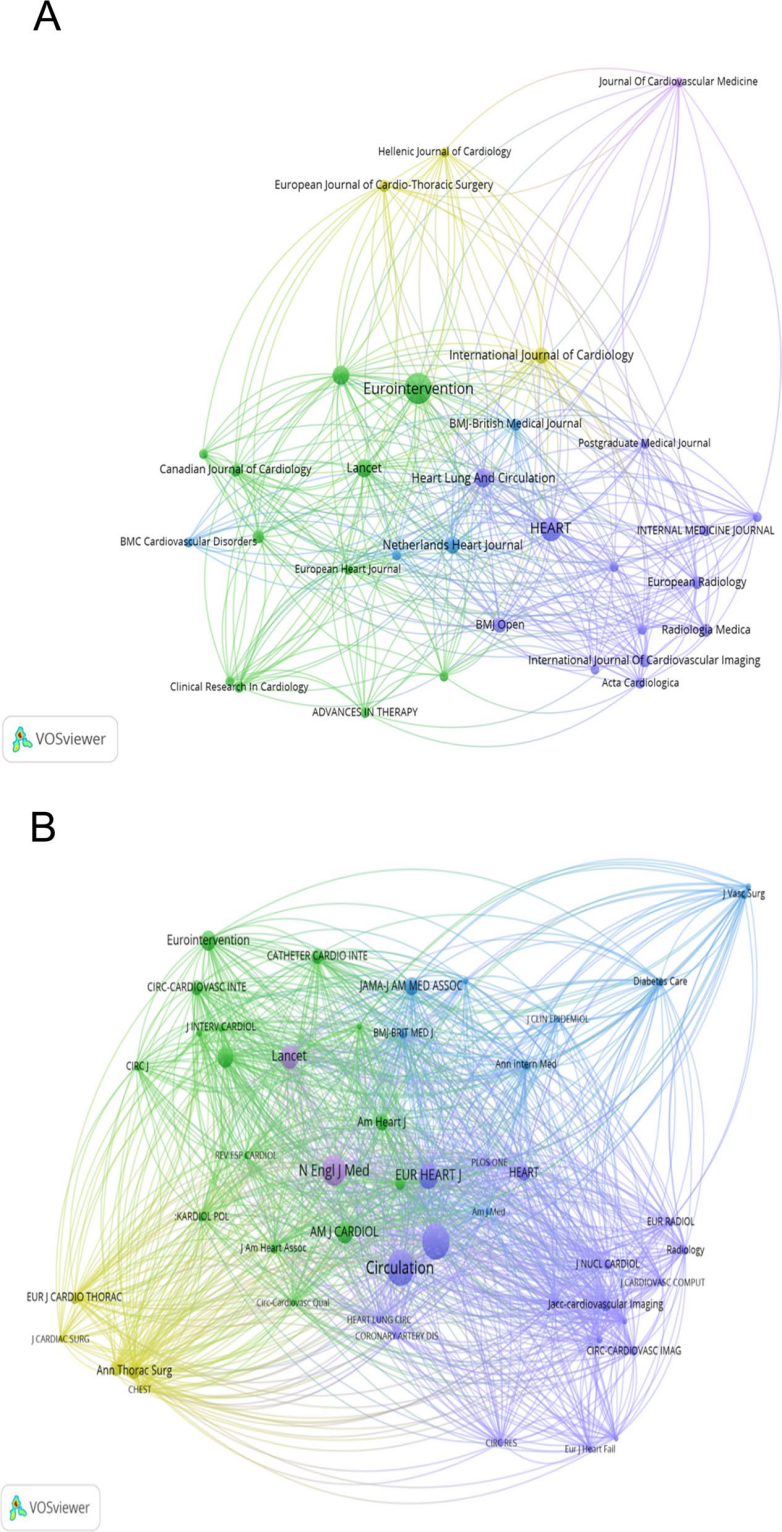
Journal bibliographic coupling network and co-citation analysis. (**A**) The bibliographic coupling network of journals. (**B**) The co-citation network visualization of journals with a minimum of 25 citations

The 372 publications were distributed across 95 journals. *EuroIntervention* published the most articles (n = 104), reflecting its niche focus. In contrast, high-impact general medical journals like The Lancet (n = 17) and The New England Journal of Medicine (despite fewer publications) accrued the highest total and per-paper citation counts (Fig. 5B), highlighting their role in disseminating landmark trials that define practice. Journal bibliographic coupling (Fig. 5A) shows clusters encompassing interventional cardiology, general cardiology, and cardiovascular imaging journals, illustrating the multidisciplinary engagement with revascularization research (Table 4).

**Table 4:** Top 20 journals in the research field

Rank	Journals	Citations	Publications	Average Citation/Publication
1	Eurointervention	2753	104	26.47
2	Heart	1050	46	22.82
3	Heart Lung and Circulation	163	21	7.76
4	Kardiologia Polska	143	19	7.53
5	Lancet	3722	17	218.94
6	Netherlands Heart Journal	65	14	4.64
7	International Journal of Cardiology	113	10	11.30
8	BMJ Open	63	9	7.00
9	European Heart Journal -Acute Cardiovascular Care	61	5	12.20
10	European Radiology	70	5	14.00
11	International Journal of Cardiovascular Imaging	26	5	5.20
12	Radiologia Medica	66	5	13.2
13	Bmj-british Medical Journal	435	4	108.75
14	Canadian Journal of Cardiology	9	4	2.25
15	European Journal of Cardio-thoracic Surgery	161	4	40.25
16	Clinical Research in Cardiology	38	3	12.67
17	Acta Cardiologica	39	3	13.00
18	Internal Medicine Journal	23	3	7.67
19	European Heart Journal	269	2	134.50
20	Clinical Medicine	3	2	1.50

### Knowledge base and foundational references

**Fig. 6:**
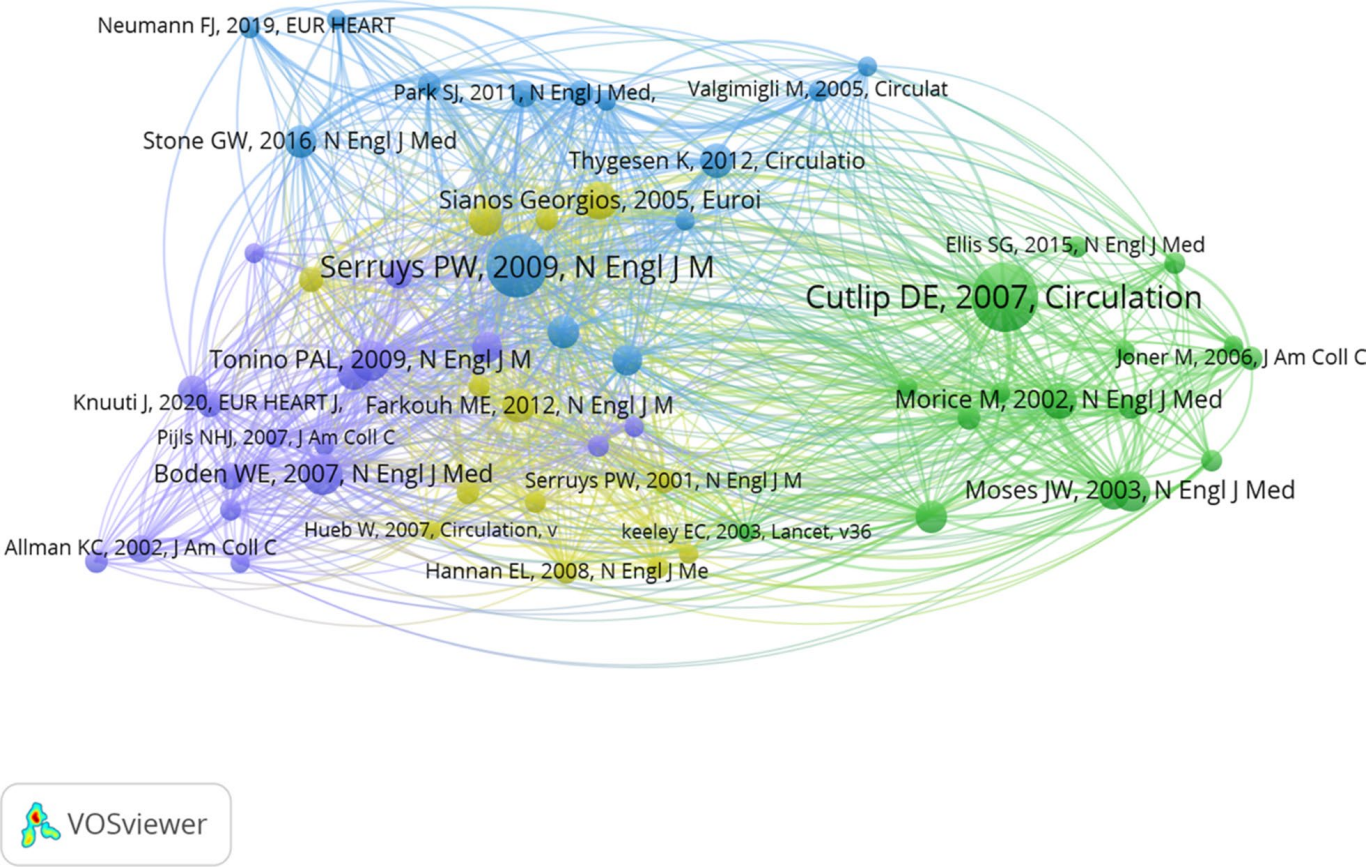
The co-citation network visualization of references with a minimum of 10 citations

The dataset cited 9,221 unique references. Fig. 6 visualizes the co-citation network of references cited 10 or more times, where node size corresponds to citation frequency. Table 5 lists the top 10 co-cited references. The most influential was the consensus paper by Cutlip et al. on standardized clinical endpoint definitions, a cornerstone for trial design. Other foundational works include pivotal trials comparing PCI with CABG (SYNTAX), assessing fractional flow reserve (FAME), and evaluating DES efficacy. Citation burst detection (Supplementary Figure S4) showed that bursts of attention consistently followed the publication of major trial results and safety debates (e.g., late stent thrombosis).

**Table 5:** Top 10 highly cited references in the research field

Rank	Cocitations	Title	Author	Journal	Published year	Country
1	66	Clinical end points in coronary stent trials: a case for standardized definitions	Donald E Cutlip	Circulation	2007	USA
2	58	Percutaneous coronary intervention versus coronary-artery bypass grafting for severe coronary artery disease	Patrick W Serruys	The New England Journal of Medicine	2009	Netherlands
3	30	Optimal medical therapy with or without PCI for stable coronary disease	William E Boden	The New England Journal of Medicine	2007	USA
4	28	Sirolimus-eluting stents versus standard stents in patients with stenosis in a native coronary artery	Jeffrey W Moses	The New England Journal of Medicine	2003	USA
5	28	Fractional flow reserve versus angiography for guiding percutaneous coronary intervention	Pim A L Tonino	The New England Journal of Medicine	2009	Netherlands
6	26	The SYNTAX Score: an angiographic tool grading the complexity of coronary artery disease	Georgios Sianos	EuroIntervention	2005	Netherlands
7	25	A randomized comparison of a sirolimus-eluting stent with a standard stent for coronary revascularization	Marie-Claude Morice	The New England Journal of Medicine	2002	France
8	23	Coronary artery bypass graft surgery versus percutaneous coronary intervention in patients with three-vessel disease and left main coronary disease: 5-year follow-up of the randomised, clinical SYNTAX trial	Friedrich W Mohr	Lancet	2013	Germany
9	23	Strategies for multivessel revascularization in patients with diabetes	Michael E Farkouh	The New England Journal of Medicine	2012	USA
10	22	Fractional flow reserve-guided PCI versus medical therapy in stable coronary disease	Bernard De Bruyne	The New England Journal of Medicine	2012	Belgium

### Keywords analysis and thematic clusters

**Fig. 7:**
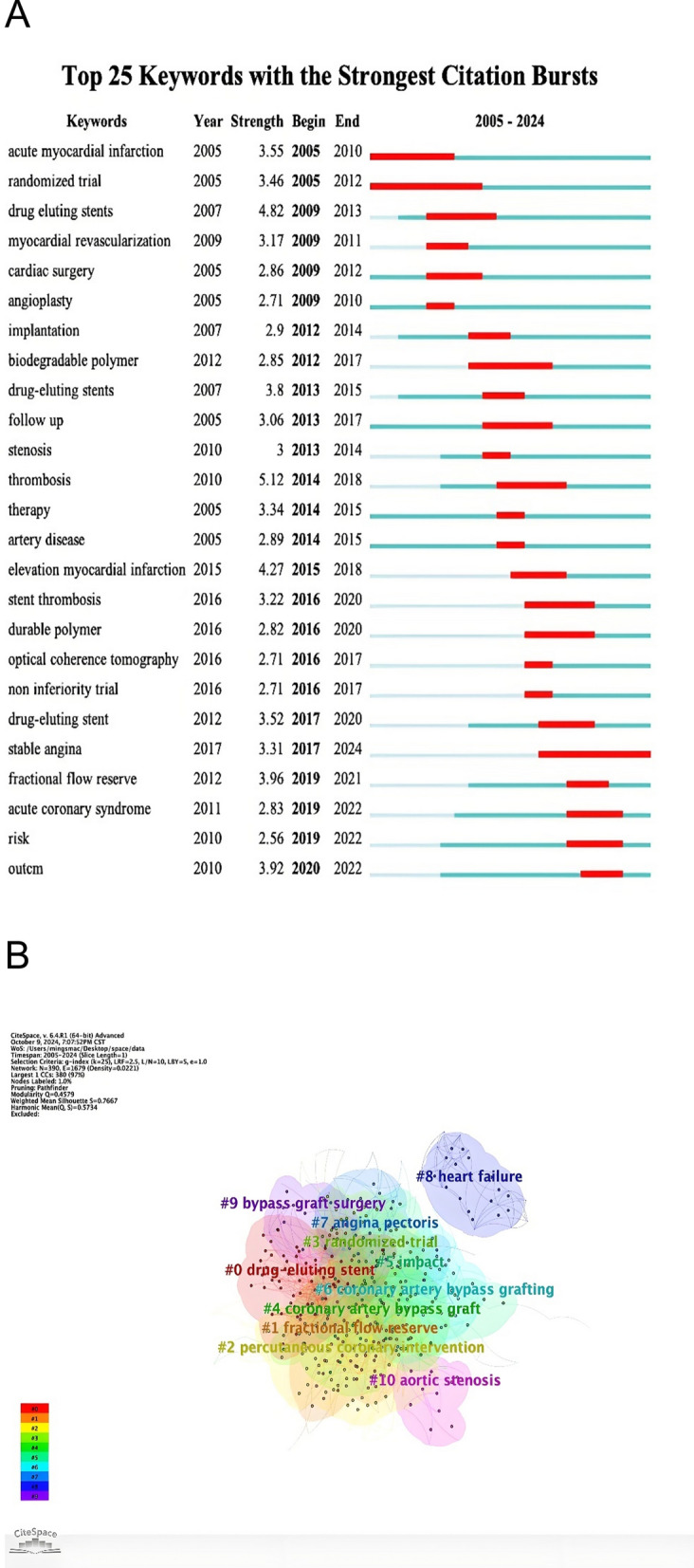
Keywords burst detection and clustering mapping analysis. (**A**) Burst analysis of the top 25 keywords. (**B**) Keyword clustering map analysis through Cite Space

Keyword analysis identified 19,221 occurrences, with the 30 most frequent terms presented in Supplementary Table 1 and Supplementary Figure S5A. The most frequent keywords were “drug-eluting stents,” “thrombosis,” “myocardial infarction,” and “fractional flow reserve.” Co-occurrence network analysis and CiteSpace clustering (Fig. 7B) identified several major thematic clusters: Cluster 1 (DES Technology & Safety)—Centered on stent thrombosis, polymer technology, and long-term safety; Cluster 2 (Physiological Guidance)—Focused on fractional flow reserve (FFR) and ischemia-guided revascularization; Cluster 3 (Acute Coronary Syndromes)—Encompassing STEMI, complete revascularization, and antiplatelet therapy.

It is noteworthy that keywords and clusters directly related to surgical revascularization (CABG) were minimally represented, indicating a pronounced literature focus on PCI within our retrieved dataset.

### Thematic evolution and emerging frontiers

Burst detection of the top 25 keywords (Fig. 7A**)** traces the field’s dynamic focus: early bursts on “sirolimus-eluting stents” and “thrombosis” (2007–2012) gave way to “fractional flow reserve” and “myocardial infarction” (2013–2018). Recent bursts (2019–2024) include “complete revascularization,” “guideline,” and “myocardial perfusion,” reflecting current emphasis on evidence-based, strategy-oriented, and functionally-guided care.

Timeline visualization of keyword clusters (Supplementary Figure S5C) confirms this evolution. The field has progressively shifted from device-specific efficacy concerns (early DES) towards patient-oriented outcome research, strategic comparisons (PCI vs. CABG, complete vs. incomplete revascularization), and integration of advanced imaging and physiological metrics to guide personalized therapy.

## Discussion

This bibliometric analysis synthesized 372 articles on coronary revascularization, published between January 2005 and September 2024 across 55 countries. Through an analysis of 372 high-impact publications, we have mapped the knowledge architecture of this pivotal cardiovascular domain, revealing its productivity patterns, core networks, intellectual foundations, and thematic evolution. Our findings characterize a mature, dynamic, and globally collaborative research ecosystem driven by landmark clinical trials and technological innovations.

### Global research productivity and collaboration patterns

The analysis revealed a steady growth in annual publications, with prominent contributions from North America, Europe, and East Asia, particularly the United States, China, and Germany. This geographic distribution aligns with the global burden of CAD, as these regions face significant cardiovascular disease mortality and have invested heavily in cardiovascular research infrastructure [15]. International collaboration emerged as a key driver of high-impact research, with the MCP ratio indicating that collaborative studies consistently achieved higher citation impact than single-institution or single-country works. This underscores the necessity of cross-border knowledge exchange in addressing complex clinical challenges such as revascularization strategy selection and long-term outcome optimization.

Notably, the concentration of top productive institutions in academic medical centers and cardiovascular specialty hospitals highlights the critical role of specialized research teams in advancing the field. These institutions often lead large-scale randomized controlled trials (RCTs) and registries that shape clinical guidelines, such as the SYNTAX, EXCEL, and NOBLE trials which established pivotal evidence for PCI versus CABG decision-making [16–18]. The author collaboration network further identified core research clusters focused on interventional cardiology, cardiac surgery, and outcomes research, reflecting the multidisciplinary nature of coronary revascularization research.

### Thematic evolution and research hotspots

Keyword co-occurrence and clustering analysis identified three dominant thematic trajectories over the study period. The first trajectory centers on the "PCI versus CABG" comparative effectiveness debate, which has evolved from early assertions of CABG superiority in multivessel and unprotected left main CAD to contemporary recognition of equipoise in selected patient subgroups [16, 19]. Recent studies, including meta-analyses and updated guidelines, emphasize individualized decision-making based on anatomical complexity (e.g., SYNTAX score II) and clinical factors such as age, diabetes, and left ventricular function [16, 20, 21]. The SYNTAX score II has been validated to predict long-term mortality even in patients with one- or two-vessel disease, expanding its utility beyond complex multivessel cases [16]. For patients with diabetes and multivessel disease, pooled analysis of data from the SYNTAX, PRECOMBAT, and BEST trials confirmed the value of SYNTAX scores I and II in guiding revascularization choices [16].

The second key theme is the advancement of minimally invasive revascularization techniques, including minimally invasive coronary artery bypass grafting (MIDCAB), totally endoscopic coronary artery bypass (TECAB), and hybrid coronary revascularization [22]. These approaches address the trade-off between the clinical benefits of CABG and patient preferences for less invasive procedures, aiming to reduce sternotomy-related complications while maintaining revascularization efficacy. Based on recent high-quality studies directly comparing postoperative recovery and clinical outcomes between minimally invasive coronary artery bypass grafting (CABG) and conventional sternotomy CABG, current evidence indicates that minimally invasive CABG is feasible in specific patient populations. Furthermore, it may offer potential advantages in reducing early postoperative pain, shortening mechanical ventilation time, decreasing hospital length of stay, and enhancing recovery in certain physical function metrics, such as early mobility. [23]. Despite barriers to widespread adoption such as technical complexity and cost, emerging evidence supports their safety and potential to redefine surgical revascularization paradigms.

The third prominent theme reflects technological innovation in interventional practice, including intravascular imaging (OCT/IVUS), functional assessment (FFRangio), and AI-assisted plaque analysis [24–26]. Recent trials like OCT-FUJI have demonstrated the utility of high-resolution imaging in optimizing stent implantation and reducing adverse outcomes, while AI-driven tools show promise in identifying high-risk plaques and guiding personalized intervention strategies [24, 25]. The development of next-generation devices such as bioadaptable stents further exemplifies the integration of engineering and clinical science to improve long-term PCI outcomes [27]. Second-generation drug-eluting stents have also shown favorable two-year outcomes in patients with multivessel disease and left main stenosis, contributing to the evolving interventional toolkit [28].

### Implications for clinical practice and future research

The bibliometric findings have direct clinical implications. The dominance of comparative effectiveness research reinforces the role of heart team collaboration in treatment decision-making, as recommended by current guidelines. The SYNTAX score II, which integrates clinical and anatomical variables to predict mortality with different revascularization strategies, has emerged as a practical tool to implement this personalized approach [20, 21]. The 1-year results of the SYNTAX II study further validated state-of-the-art percutaneous revascularization in patients with de novo three-vessel disease, supporting its clinical utility [18].

Technological innovations highlighted in the thematic analysis are rapidly translating into clinical practice. OCT-guided PCI is increasingly preferred for complex lesions and acute coronary syndromes due to its superior resolution in detecting stent malapposition and plaque characteristics [24]. AI-assisted plaque analysis and non-invasive functional assessment tools like FFRangio address unmet needs in risk stratification and avoiding unnecessary revascularization, particularly in resource-limited settings [25, 26]. For patients with chronic kidney disease, who face elevated coronary event risk, these tools may also enhance risk stratification and treatment personalization [29].

Future research priorities should focus on several gaps identified in the current landscape. First, long-term comparative data on minimally invasive techniques and novel stenting platforms are needed to confirm their durability and cost-effectiveness [27]. Second, studies addressing health disparities in revascularization access and outcomes, particularly in low- and middle-income countries, are urgently required given the shifting global CAD burden [15, 30]. Third, integration of digital health tools (e.g., AI prediction models, remote monitoring) into revascularization care pathways represents a promising direction to improve patient-centered outcomes.

### Limitations

This study is the first to apply bibliometric methods to coronary revascularization. Nonetheless, several limitations should be acknowledged.

First, database and language biases may limit comprehensiveness. The analysis relied solely on the Web of Science Core Collection (WoSCC), potentially omitting relevant studies from other databases (e.g., PubMed Central, Scopus) or regional repositories. Additionally, only English-language publications were included, which may underrepresent contributions from non-English-speaking regions. While this aligns with standard bibliometric practices for data consistency [31, 32], it should be noted that the global research landscape presented may not be fully exhaustive.

Second, inherent biases in bibliometric indicators exist. Citation counts, journal impact factors, and publication volume—core metrics in this study—have inherent limitations: citation patterns favor English-language/Western studies and high-visibility research (e.g., large RCTs), while publication volume alone does not reflect research quality, methodological rigor, or real-world clinical impact (e.g., guideline-informing studies with lower citation rates). Furthermore, indexing delays for publications in 2024 (up to September) may have underestimated recent emerging trends (e.g., AI-assisted revascularization), as WoSCC typically requires 1–3 months for full indexing [33, 34].

Third, the influence of hyper-prolific authors was not addressed via sensitivity analysis. A small number of hyper-prolific authors (e.g., Serruys PW, Stone GW) may disproportionately affect country/institutional rankings and collaboration networks. We did not exclude these authors because their work represents landmark advances that have shaped the field, and exclusion could obscure the true structure of influential research networks. To mitigate potential bias, our analysis complemented publication volume with multiple indicators (e.g., average citations per publication, collaboration density), reducing overreliance on single metrics.

Future bibliometric efforts should incorporate multiple databases and languages, integrate alternative metrics (e.g., altmetrics, guideline citations), and perform sensitivity analyses for a broader perspective.

## Conclusion

This study provides a comprehensive bibliometric mapping of global research trends in coronary revascularization over the past two decades (2005–2024). By analyzing publication patterns, collaborative networks, and intellectual structures, we delineate a dynamic field characterized by sustained productivity, deep international collaboration, and a clear evolution from device-centric innovation towards strategy-oriented and personalized patient management. Our analysis identifies leading national and institutional contributors, foundational clinical trials, and core journals that have shaped the evidence base. The thematic progression—from early focus on drug-eluting stent safety, through the era of physiological guidance, to current priorities in complete revascularization and guideline implementation—reflects the field’s maturation in aligning technological advances with patient-centered outcomes.

Beyond a retrospective summary, this bibliometric map serves as a strategic tool. It provides clinicians and trainees with a structured overview of the field’s knowledge architecture, helps researchers identify influential networks and emerging frontiers, and offers funders insight into the collaborative engines that drive high-impact evidence. The observed under-representation of surgical (CABG) themes within the keyword landscape highlights an opportunity for more balanced bibliometric visibility in future research synthesis.

Ultimately, this work establishes a foundational reference point for quantifying and visualizing the scientific discourse in coronary revascularization. Future analyses can build upon this map to track the development of emerging frontiers, such as the integration of artificial intelligence, long-term outcomes of novel devices, and refined risk-stratification models, ensuring that the evolution of this critical field continues to be guided by robust, collaborative, and patient-focused science.

## Supplementary Information

Supplementary Material 1

Supplementary Material 2

## Data Availability

The raw data supporting the conclusions of this article will be made available by the authors, without undue reservation.
