# Supplementary material for: Global trends and research hotspots in coronary revascularization for ischemic heart disease: a bibliometric analysis (2005–2024)
Source: J Cardiothorac Surg. 2026 Mar 3;21:176. doi: 10.1186/s13019-026-03937-w (PMC13067637; doi:10.1186/s13019-026-03937-w)
Supplement: Supplementary file 1 — Supplementary Material 1 [file 13019_2026_3937_MOESM1_ESM.docx]

Supplementary Table 1. The top 30 keywords in the ICM research field.

| Rank | Keywords | Occurrences |
| --- | --- | --- |
| 1 | Intervention | 72 |
| 2 | outcm | 65 |
| 3 | revascularization | 64 |
| 4 | follow up | 52 |
| 5 | coronary artery disease | 50 |
| 6 | artery disease | 47 |
| 7 | angioplasty | 42 |
| 8 | disease | 32 |
| 9 | implantation | 32 |
| 10 | surgery | 32 |
| 11 | thrombosis | 32 |
| 12 | metaanalysis | 29 |
| 13 | impact | 28 |
| 14 | bare metal stents | 26 |
| 15 | myocardial infarction | 26 |
| 16 | clinical outcm | 26 |
| 17 | angiography | 26 |
| 18 | fractional flow reserve | 25 |
| 19 | trial | 23 |
| 20 | acute myocardial infarction | 23 |
| 21 | randomized trial | 23 |
| 22 | risk | 22 |
| 23 | mortality | 22 |
| 24 | stenosis | 20 |
| 25 | bypass graft surgery | 19 |
| 26 | multicenter | 19 |
| 27 | percutaneous coronary intervention | 18 |
| 28 | management | 15 |
| 29 | association | 15 |
| 30 | survival | 14 |
