# Supplementary material for: Global trends and research hotspots in coronary revascularization for ischemic heart disease: a bibliometric analysis (2005–2024)
Source: J Cardiothorac Surg. 2026 Mar 3;21:176. doi: 10.1186/s13019-026-03937-w (PMC13067637; doi:10.1186/s13019-026-03937-w)
Supplement: Supplementary file 2 — Supplementary Material 2 [file 13019_2026_3937_MOESM2_ESM.docx]

Title/abstract screening

Exclude records that are not published between 2005.1.1-2024.9.30; non-English,non-original articles and reviews (e.g., editorials, letters to the editor, case reports); and irrelevant topics (e.g., revascularization for non-coronary vascular diseases)

First screening: retrieved from Web of Science Core Collection on October 1,2024 with search strategy TS = (“myocardial ischemia” OR “coronary artery disease” OR “ischemia”) AND TS = (“revascularization”)

Remove duplicates (WoSCC tool + manual verification)

Exclude 153 records of early access (not fully indexed), complete data (not-extract author/institution/key words), focus on N-CAD population (e.g peripheral vascular disease, congenital heart disease) or only basic experiments

Full-text screening

Steps

Supplementary FigureS1. Flow chart of the publication filtering process.

Supplementary Figure S2. Histogram of top 10 productive countries.

Supplementary Figure S3A. Histogram of top 13 productive authors.


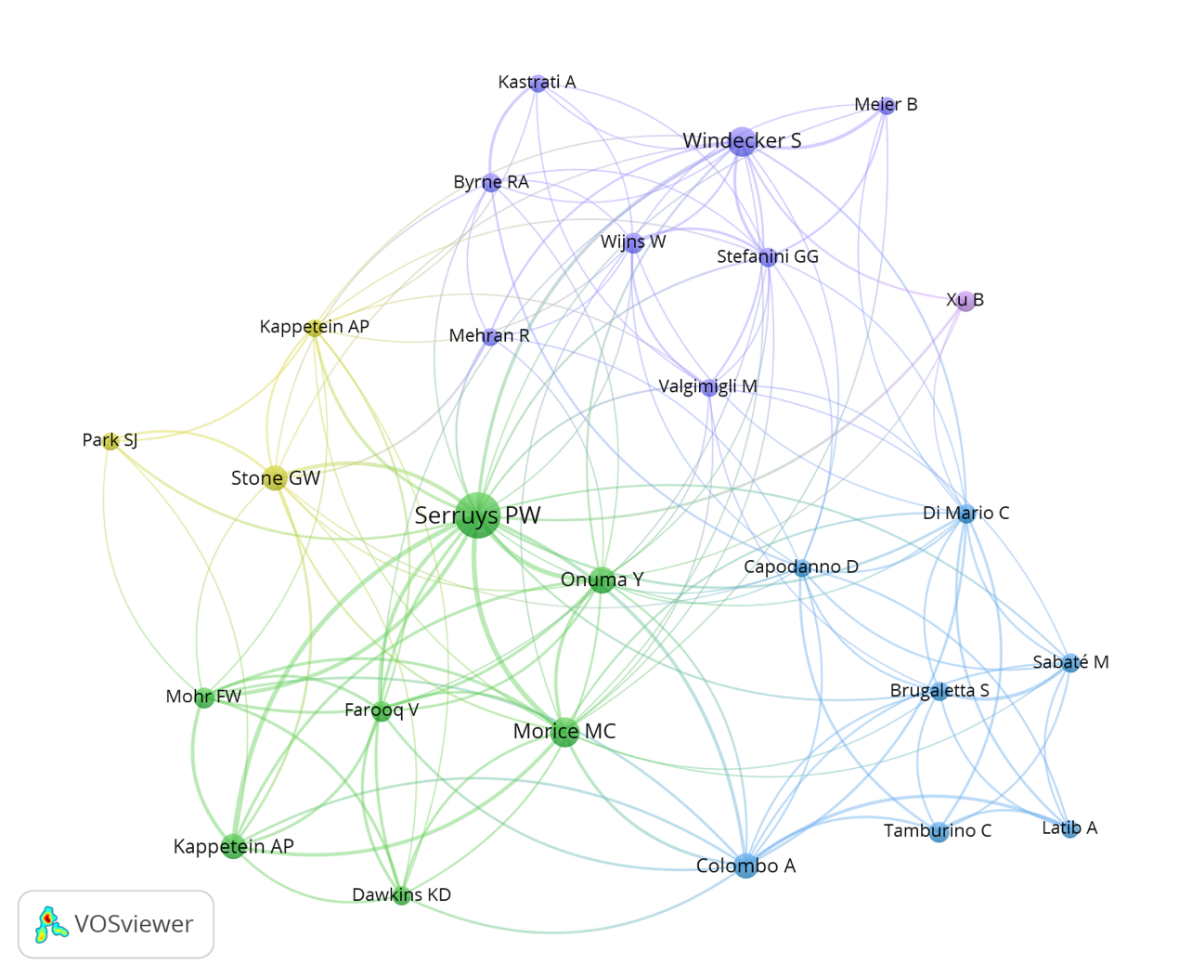


Supplementary Figure S3B. Co-occurrence map of authors.


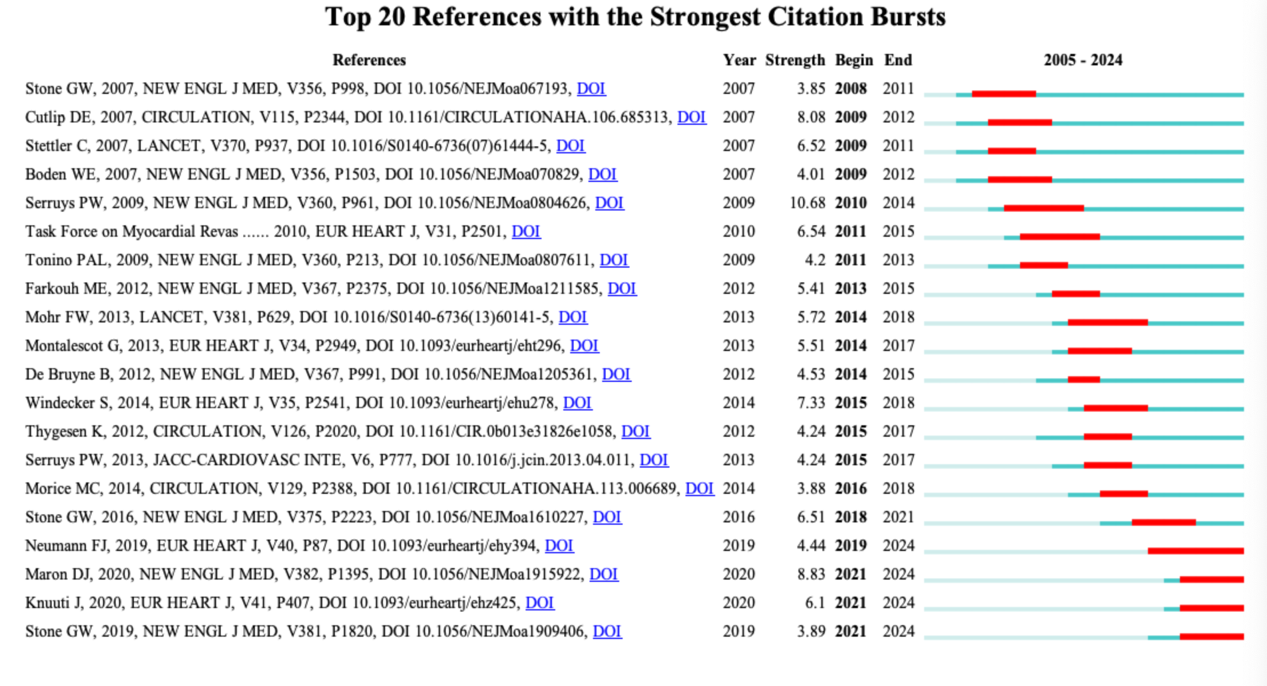


Supplementary Figure 4. Burst analysis of the top 20 references.


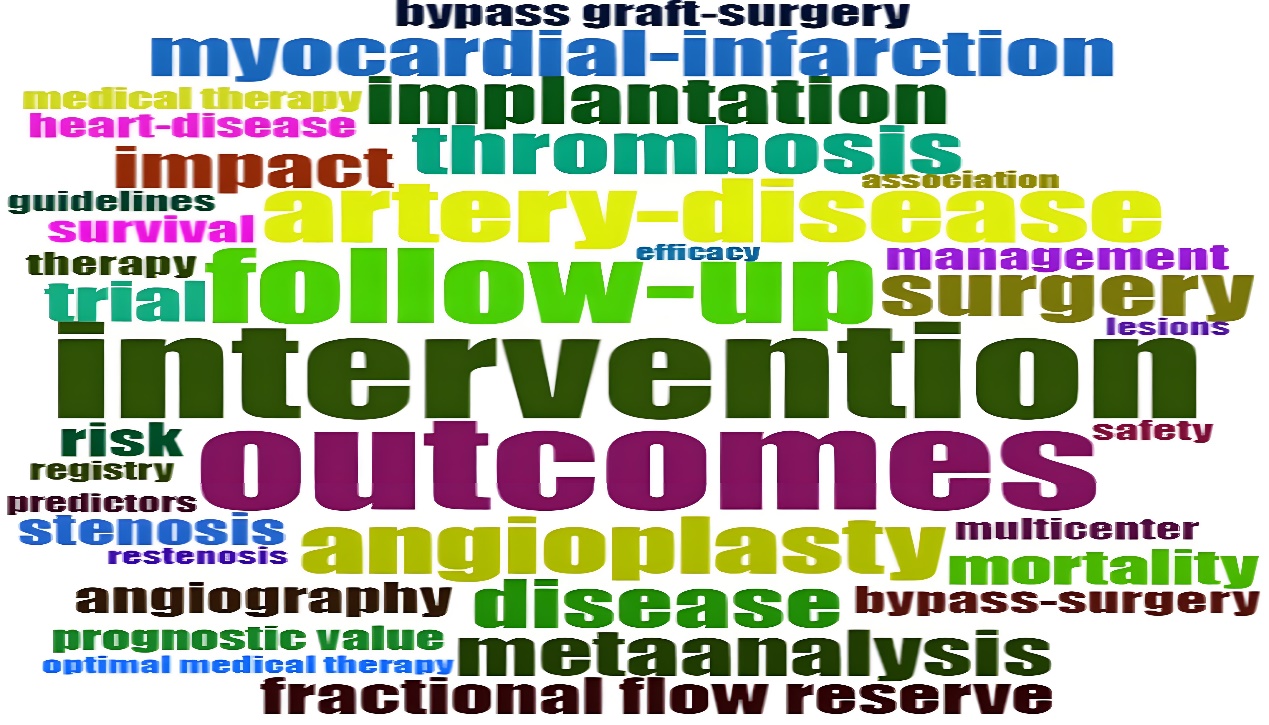


Supplementary Figure S5A. Keywords tree graph.


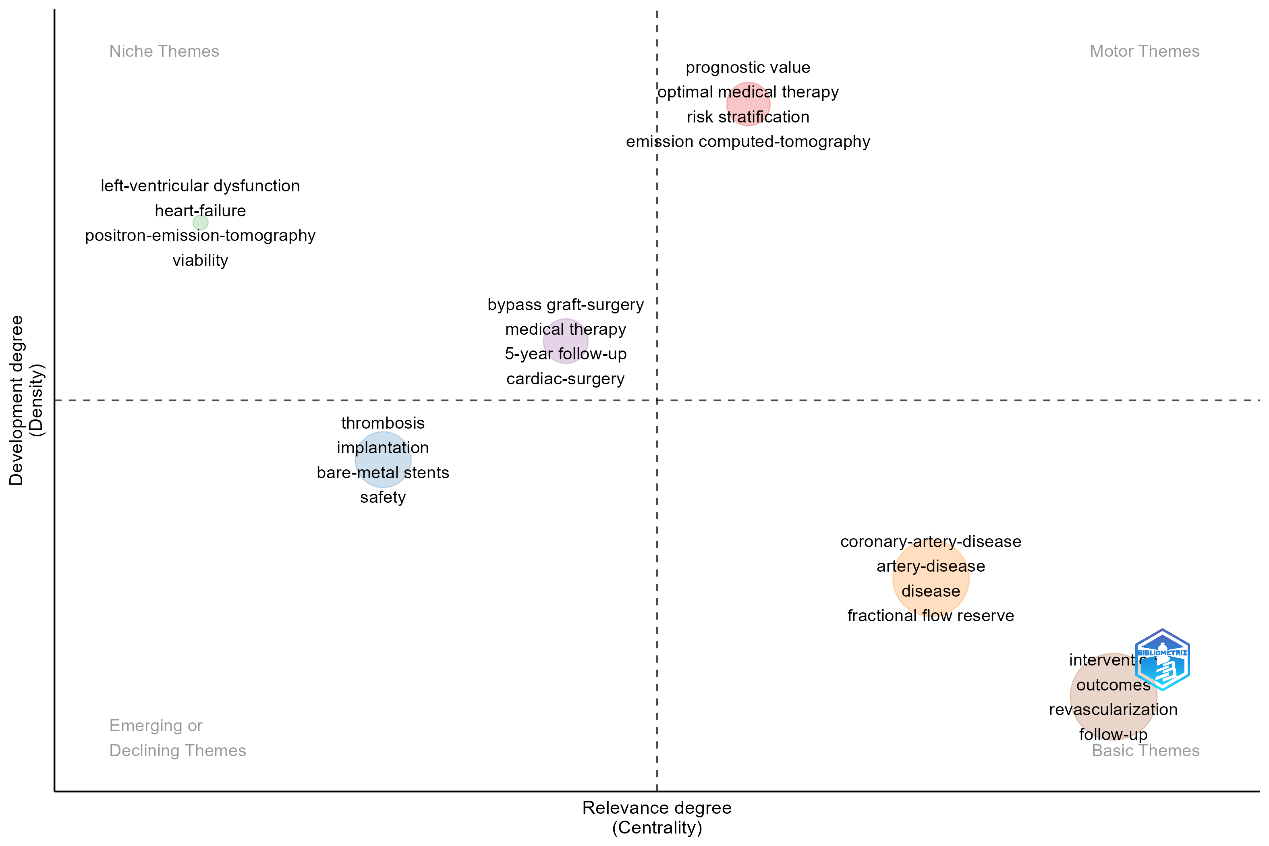


Supplementary Figure S5B.


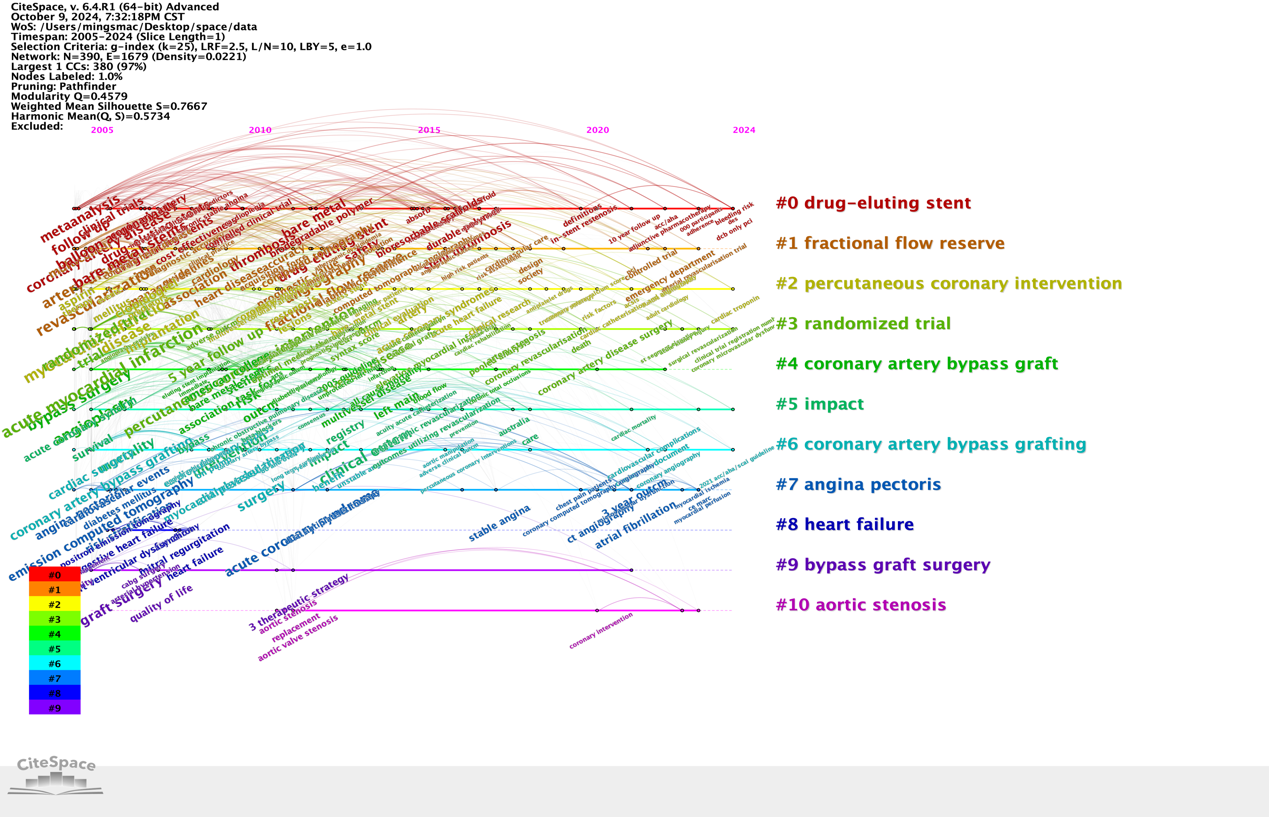


Supplementary Figure S5C. The timeline view for keywords.
